# Merging traditional practices and modern technology through computational plant breeding

**DOI:** 10.1093/plphys/kiaf355

**Published:** 2025-08-08

**Authors:** Mohsen Yoosefzadeh-Najafabadi

**Affiliations:** Department of Plant Agriculture, University of Guelph, Guelph, ON N1G 2W1, Canada

## Abstract

Plant breeding has transitioned from its ancient agrarian roots to a modern, sophisticated discipline blending advanced genetic and computational techniques. Initially led by intuition and basic selection, the field was revolutionized in the 19th century by Gregor Mendel's principles. Today, plant breeding utilizes multiomics approaches and data science techniques to navigate vast amounts of data and deepen our understanding of the biological mechanisms behind specific traits. To tackle the challenges of big data, the discipline now incorporates computational biology, data science, and bioinformatics, which have become integral to routine plant breeding practices. As plant breeders have explored these promising fields, many have adopted titles such as “plant breeder and computational biologist” or “plant breeder and bioinformatician.” However, these titles may lead to misconceptions about expertise, as breeders often apply a blend of these skills without specializing fully in each domain. Recognizing this, it is crucial to establish a clear identity for the evolving skill set of modern plant breeders. In this review, I explore the historical evolution of plant breeding, highlighting the transformative role of computational biology. Furthermore, I address the potential pitfalls of adding titles to plant breeding and propose the adoption of the term “computational plant breeding.” This term more accurately reflects the integrated application of computational tools and biological insights in plant breeding. By redefining this emerging field, we can better appreciate its unique contributions and prepare for future advancements in agricultural science.

## Teaser

Computational plant breeding combines data, biology, and digital tools to accelerate crop improvement and address growing agricultural demands in a rapidly changing world.

## Introduction

Plant breeding has been foundational to the advancement of human civilization for over 10,000 years, moving from the instinctive selection of desirable traits (e.g. yield, taste, and harvestability) to recent advanced scientific methodologies (Vaughan et al. 2007; DeHaan et al. 2016). A full list of crops that were domesticated in different regions worldwide is provided in Table 1. A major leap in plant breeding occurred in the 19th century when Gregor Mendel established the laws of inheritance, an event considered to represent the foundation of modern plant breeding and genetics (Berry and Browne 2022). In the 20th century, revolutionary advances, including hybridization, the development of tissue culture methods, and the Green Revolution, increased agricultural productivity globally and mitigated food insecurity, especially in developing countries (Kumar 2022). The integration of genetics and molecular biology opened the area of genetic engineering and genetically modified organisms (GMOs), although not without inciting debates over biosafety (Krimsky 2019). In the new millennium, multiomics approaches, including whole-genome sequencing, high-throughput phenotyping (phenomics), transcriptomics, and other omics are providing plant breeders with the ability to precisely select and advance large breeding populations to produce high-performing, resilient crop varieties that are better suited to meet modern agricultural challenges (Yoosefzadeh-Najafabadi and Rajcan 2023).

**Table 1.** Estimated ages and species names of domesticated plants across different regions worldwide

Region	Plant species	Species name	Age	References
Middle East	Wheat	*Triticum* spp.	10,000	Charmet (2011)
	Barley	*Hordeum vulgare*	10,000	Badr et al. (2000)
	Lentils	*Lens culinaris*	9,500	Ambika et al. (2022)
	Chickpeas	*Cicer arietinum*	9,500	Igolkina et al. (2023)
East Asia	Rice	*Oryza sativa*	7,000	Cox (2009)
	Soybean	*Glycine max*	4,000	Cox (2009)
South Asia	Mung Beans	*Vigna radiata*	4,000	Huppertz et al. (2023)
	Black Gram	*Vigna mungo*	4,500	Verma et al. (2022)
Southeast Asia	Taro	*Colocasia esculenta*	10,000	Ahmed et al. (2020)
	Bananas	*Musa* spp.	7,000	Cox (2009)
Africa	Sorghum	*Sorghum bicolor*	8,000	Cox (2009)
	Millet	*Setaria italica*	8,000	Cox (2009)
Europe	Oats	*Avena sativa*	3,000	Kamal et al. (2022)
	Rye	*Secale cereale*	5,000	Behre (1992)
Mesoamerica	Maize (Corn)	*Zea mays*	7,500	Cox (2009)
	Common Beans	*Phaseolus* spp.	7,500	Cox (2009)
	Squash	*Cucurbita* spp.	10,000	Smith (2001)
South America	Potatoes	*Solanum tuberosum*	4,500	Cox (2009)
	Quinoa	*Chenopodium quinoa*	7,000	Bazile et al. (2016)
North America	Sunflower	*Helianthus annuus*	3,000	Cox (2009)

Age: approximate age of oldest evidence of domestication (years ago).

Alongside advancements in plant breeding, statistical tools have played a transformative role, evolving from performing basic assessments to conducting sophisticated multifactorial analyses describing complex trait inheritance (Mansoor et al. 2024). The contributions of pioneers such as Ronald A. Fisher were pivotal in embedding statistical methodologies within genetics and developing models to quantify genetic variance and heritability, which are key elements in successful breeding strategies (Kennedy-Shaffer 2024). As the skills of breeders advance, they increasingly rely on diverse data from various omics, each with unique data points and properties, requiring the use of advanced statistical and computational methods (Yoosefzadeh-Najafabadi et al. 2024). For example, unlike human or mammalian genomes, which are typically diploid and relatively compact (e.g. ∼3 Gb for humans), plant genomes often exhibit vast sizes (e.g. wheat at ∼17 Gb) and varying ploidy levels (e.g. diploid rice, hexaploid wheat), complicating sequencing, assembly, and annotation efforts. These characteristics demand advanced computational tools beyond those designed for smaller genomes, as traditional bioinformatics approaches struggle to handle the scale and complexity of plant genomic data. Alongside these advancements, statistical tools have evolved from performing basic assessments to conducting sophisticated multifactorial analyses of complex trait inheritance (Mansoor et al. 2024), further underscoring the need for bioinformatics and computational biology to unlock the potential of plant genomes for breeding.

As plant breeding relies heavily on data from multidisciplinary sources, the adoption of computational tools has become indispensable for modern breeders (Thriveni et al. 2024). Recognizing this need, there has been a strong emphasis on equipping future plant breeders with expertise in various data analysis techniques. In this transformative shift, fields and terms such as “computational biology” and “bioinformatics” have become integral to breeding practices. Computational biology involves the use of mathematical models, algorithms, and computational methods to understand, model, and analyze complex biological systems and interactions (Bartocci and Lió 2016). Meanwhile, bioinformatics, a subdiscipline of computational biology, is dedicated to developing methods and software tools for storing, retrieving, and analyzing vast amounts of omics data (Ma et al. 2020). The integration of these fields not only enhances the efficiency and precision of plant breeding programs but also enables breeders to harness vast datasets, accelerating the development of crop varieties that are better suited to meet evolving agricultural and environmental challenges.

In recent years, plant breeders have increasingly turned to experts in computational biology and bioinformatics for support to enhance their breeding programs (van Dijk et al. 2021). Initially, plant breeders relied heavily on these professionals, but over time, many breeders have independently acquired expertise in these fields, integrating this knowledge to advance their efforts. As a result, some plant breeders now use titles such as “plant breeder and computational biologist” or “plant breeder and bioinformatics specialist.” This practice has led to the frequent and sometimes interchangeable use of the terms “computational biology” and “bioinformatics” within the plant breeding community, despite their distinct meanings and implications. Such imprecise usage has caused confusion and misunderstanding regarding the methodologies, responsibilities, and expertise involved in plant breeding.

For a plant breeder, it is crucial to leverage the strengths of both computational biology and bioinformatics to enhance the precision of their selections and accelerate breeding programs (van Dijk et al. 2021). Plant breeders are increasingly developing various libraries, packages, and programs through extensive coding to facilitate the analysis of specific datasets, a domain primarily associated with bioinformatics (van Dijk et al. 2021; Yoosefzadeh-Najafabadi and Rajcan 2023). These tools and methods are then integrated with other strategies to interpret and deepen our understanding of the biological factors behind a trait of interest, which falls under computational biology. Consequently, titles like “plant breeder and computational biologist” or “plant breeder and bioinformatics specialist” might not fully capture the breadth of their work. A more fitting term, such as “computational plant breeding,” could more accurately describe professionals in this field. Therefore, the aim of this review is to describe the history of plant breeding, highlighting the importance of statistical methods and the need for advanced computational techniques for breeders. Moreover, this review discusses the need for precise terminology rather than data science, computational biology, and bioinformatics in plant breeding, elaborates on computational breeding terms, and describes ongoing computational breeding efforts in this domain.

## History of plant breeding

Plant breeding is an ancient practice that began over 10,000 years ago, when Neolithic people transitioned from being nomadic hunters and gatherers to members of agricultural societies (Cox 2009). This transformative shift marked the origin of plant breeding, as farmers began to selectively breed plants with desirable traits. Initially, this process was largely unconscious; farmers collected seeds from their best performing plants to sow in the next growing season (Cox 2009). Over generations, farmers ultimately domesticated staple crops, such as wheat, rice, maize, and legumes and made significant changes to traits such as yield, seed size, taste, and harvestability through natural selection and unintentional human influence (Vaughan et al. 2007; Cox 2009). The conscious manipulation of plant genetics to generate desired traits became systematic with the development of Mendelian genetics in the 19th century (Yoosefzadeh-Najafabadi et al. 2023a, 2023b, 2023c). The study of pea plants by Gregor Mendel established the fundamental laws of inheritance and provided a scientific basis for modern plant breeding. With the use of Mendel's principles, breeders were able to understand how traits were passed on from one generation to the next and were able to manipulate them by controlled crossing to generate new varieties with desirable traits (Yoosefzadeh-Najafabadi et al. 2023a, 2023b, 2023c). This period marked a significant turning point in the history of plant breeding, as breeders could now predict and control the outcomes of their breeding efforts with greater precision.

The early 20th century marked significant progress with the establishment of the concept of totipotency and the introduction of tissue culture techniques. Totipotency refers to the ability of a single cell to develop into a complete organism or to differentiate into any cell type found in that organism (Su et al. 2021). This ability allows the totipotent cell to generate all the different cell types, including somatic (body) cells and germ cells (sperm and egg), in addition to creating critical extraembryonic structures necessary for embryonic development, such as the placenta (Su et al. 2021). This concept was first demonstrated in the 1950s by F.C. Steward and his colleagues using carrot tissue (Steward et al. 1958). The discovery of totipotency improved plant breeding by giving breeders the ability to clone plant species, thus conserving their elite genotypes while avoiding genetic reshuffling by sexual reproduction. It also allowed breeders to mass propagate plants with the desired traits and enabled genetic manipulation, becoming a fundamental technique in plant biotechnology. Tissue culture involves growing plant cells, tissues, or organs in vitro under sterile conditions (Khezri et al. 2024). Tissue culture allows plants to be propagated rapidly and plays an important role in producing disease-free, high-quality planting materials, helping plant breeders overcome limitations inherent in traditional breeding methods.

The early 20th century also saw the development of hybridization techniques, which revolutionized plant breeding by creating hybrid vigor or heterosis (DeHaan et al. 2016). This was initially applied to maize by George Shull and Edward East, who demonstrated that crossing inbred lines could produce hybrid plants that significantly outperform their parents in terms of yield and resilience (Srivastava et al. 2020). This led to the widespread adoption of hybrid crops, particularly in the United States, stimulating dramatic increases in productivity and farming efficiency (Kingsbury 2009). The success of hybrid maize spurred innovations in hybrid breeding of other crops, fundamentally changing agricultural practices and food production globally (Kingsbury 2009).

The mid-20th century saw significant technological advancements and global collaborations, particularly the Green Revolution (Wu and Butz 2004). Led by Norman Borlaug, the Green Revolution involved the creation and distribution of high-yielding plants, especially in developing countries (Baranski 2022). By combining improved plant varieties with modern agricultural techniques, such as the use of fertilizers and irrigation, the Green Revolution mitigated hunger in many parts of the world while highlighting the capacity of breeding technology to help improve food security (Baranski 2022).

In the late 20th century, the discovery of DNA and advancements in molecular biology paved the way to a new era of plant breeding (Moose and Mumm 2008). Maxam–Gilbert sequencing, a technique developed by Allan Maxam and Walter Gilbert in 1977, was one of the first approaches to DNA sequencing (Gautam 2022). The Maxam–Gilbert sequencing method and Sanger sequencing, which was developed by Frederick Sanger, marked the beginning of modern genetics and genomics (Gautam 2022). Sanger sequencing, in particular, became the backbone of genetic research due to its simplicity and reliability, enabling scientists to decipher genetic codes with unprecedented accuracy (Fazendeiro and Leite 2024). These first-generation sequencing technologies provided a pathway to enhance plant breeding by producing better data, allowing plant breeders to perform detailed analyses of plant genomes and to identify genes linked with specific traits (Gautam 2022; Fazendeiro and Leite 2024).

Following these advancements in genomics, genetic engineering techniques emerged, allowing scientists to directly manipulate plant DNA (Nicholl 2023). The development of recombinant DNA technology allowed foreign genes to be introduced into plant genomes, creating GMOs. The commercialization of genetically engineered crops, such as Bt cotton and Roundup Ready soybeans, represented a paradigm shift in agricultural biotechnology, despite ongoing debates regarding their safety and environmental impact (Freeman 2012). In 1994, the Flavr Savr tomato developed by Calgene became the first genetically engineered crop to be commercialized (Kramer and Redenbaugh 1994; Kamthan et al. 2016). This tomato was engineered for delayed ripening to improve shelf life. The debut of the Flavr Savr tomato showcased the potential of genetic engineering in addressing consumer and producer needs by introducing novel traits rapidly compared with traditional breeding (Kamthan et al. 2016).

**Figure 1.**
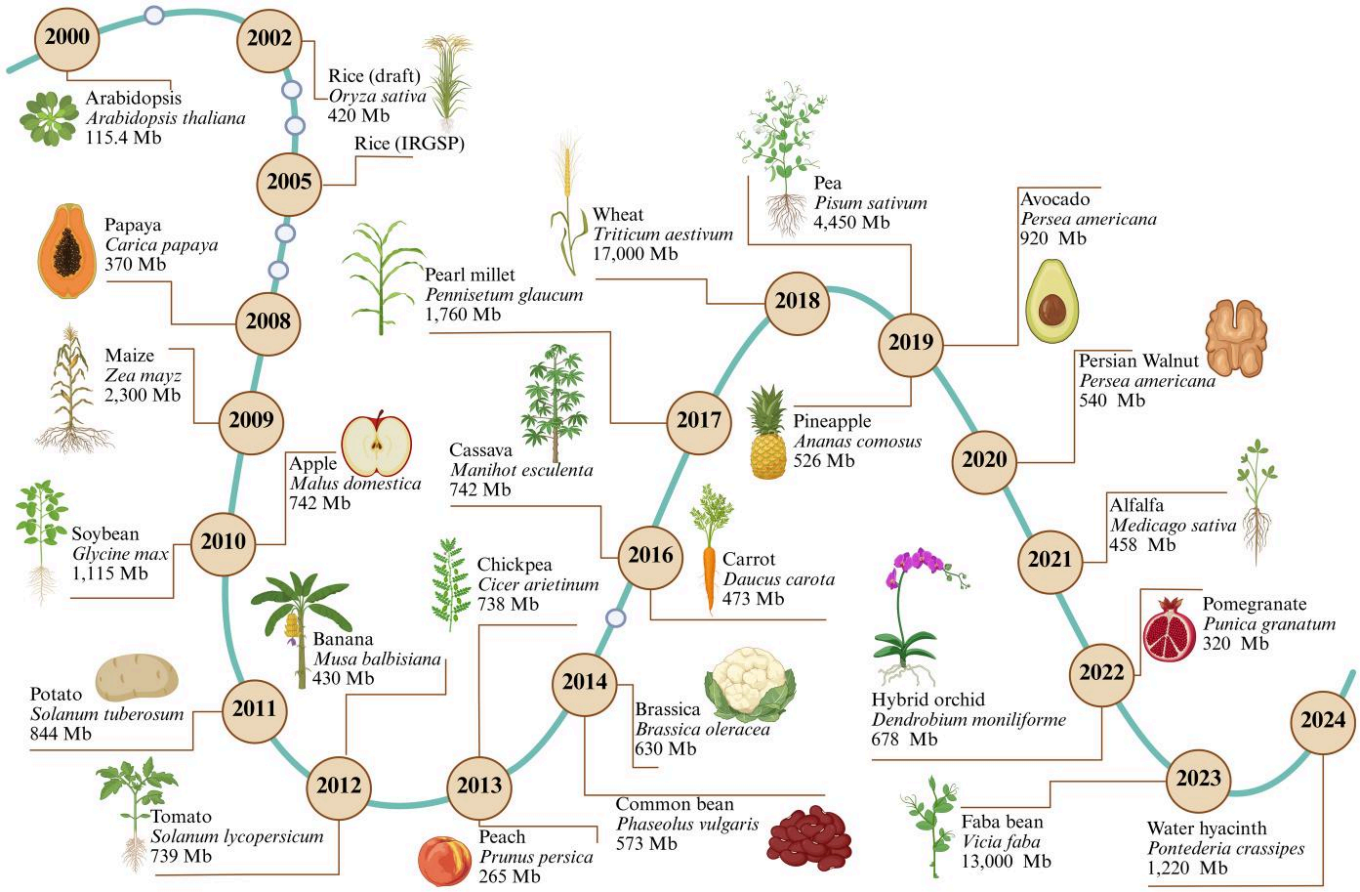
Timeline of the release of genome sequences for various plant species. Genome size (in megabases, Mb) is listed below each species name. The figure was created using BioRender.com.

The *Arabidopsis thaliana* genome, the first plant genome to be sequenced, was released in 2000, representing the first ever complete plant genomic blueprint. This information led to a deeper understanding of the structure, function, and evolution of plants (Purugganan and Jackson 2021). The use of Solexa 1G sequencing technology (later acquired by Illumina) led to a transformative shift to second-generation sequencing in the mid-2000s (Wang 2021). High-throughput sequencing technologies allowed for massively parallel sequencing, significantly reducing the cost and time required to sequence entire genomes. Illumina's sequencing platforms provide a great opportunity to study large-scale genomes through genomic analyses (Wang 2021), resulting in an exponential increase to the number of sequenced plant genomes, as summarized in Fig. 1.

Major developments in plant breeding have emerged as a result of new information and technological advancements in the 21st century, especially through improvements in genomics, bioinformatics, and sequencing. Several plant genomes have been sequenced, such as the rice, maize, and wheat genomes (Fig. 1), allowing breeders to better understand the genetic components of complex traits. Technologies such as marker-assisted selection (MAS) utilize genetic markers linked to desirable traits, enabling more efficient and targeted breeding (Yoosefzadeh-Najafabadi and Rajcan 2023). Additionally, the emergence of CRISPR–Cas9 genome editing has transformed plant breeding by offering precise and relatively rapid methods for modifying plant genomes (Chen et al. 2019). Cibus's commercialization of a herbicide-resistant canola in 2014, developed through targeted mutagenesis, marked a milestone as the first genome-edited crop (Hamburger 2019). Such advancements are considered revolutionary, promising the creation of crops with enhanced traits, such as greater nutritional value, improved pest and disease resistance, and better adaptability to environmental shifts, while also addressing public concerns about GMO safety (Hamburger 2019).

Genomic selection (GS) involves the use of genome-wide markers to predict the performance of breeding lines before they are tested in the field, thus shortening the breeding cycle (Yoosefzadeh-Najafabadi and Torkamaneh 2025). This technique, in conjunction with high-throughput phenotyping technologies that allow for the rapid and accurate measurement of plant traits, allows breeders to evaluate entire populations quickly and accurately in a large breeding population. Recent advances in omics technologies (genomics, transcriptomics, proteomics, and metabolomics) have led to a great understanding of the molecular basis of plant traits and are increasingly adopted by plant breeders (Ijaz et al. 2024). Omics technologies aid breeders by providing a holistic view of complex traits, incorporating gene–environment interactions (GEIs) and utilizing the natural diversity available in crop plants. Omics approaches facilitate both MAS and GS by identifying molecular markers associated with desirable phenotypes, ultimately leading to the targeted development of superior crop varieties (Ijaz et al. 2024).

## The role of statistics in modernizing plant breeding

**Figure 2.**
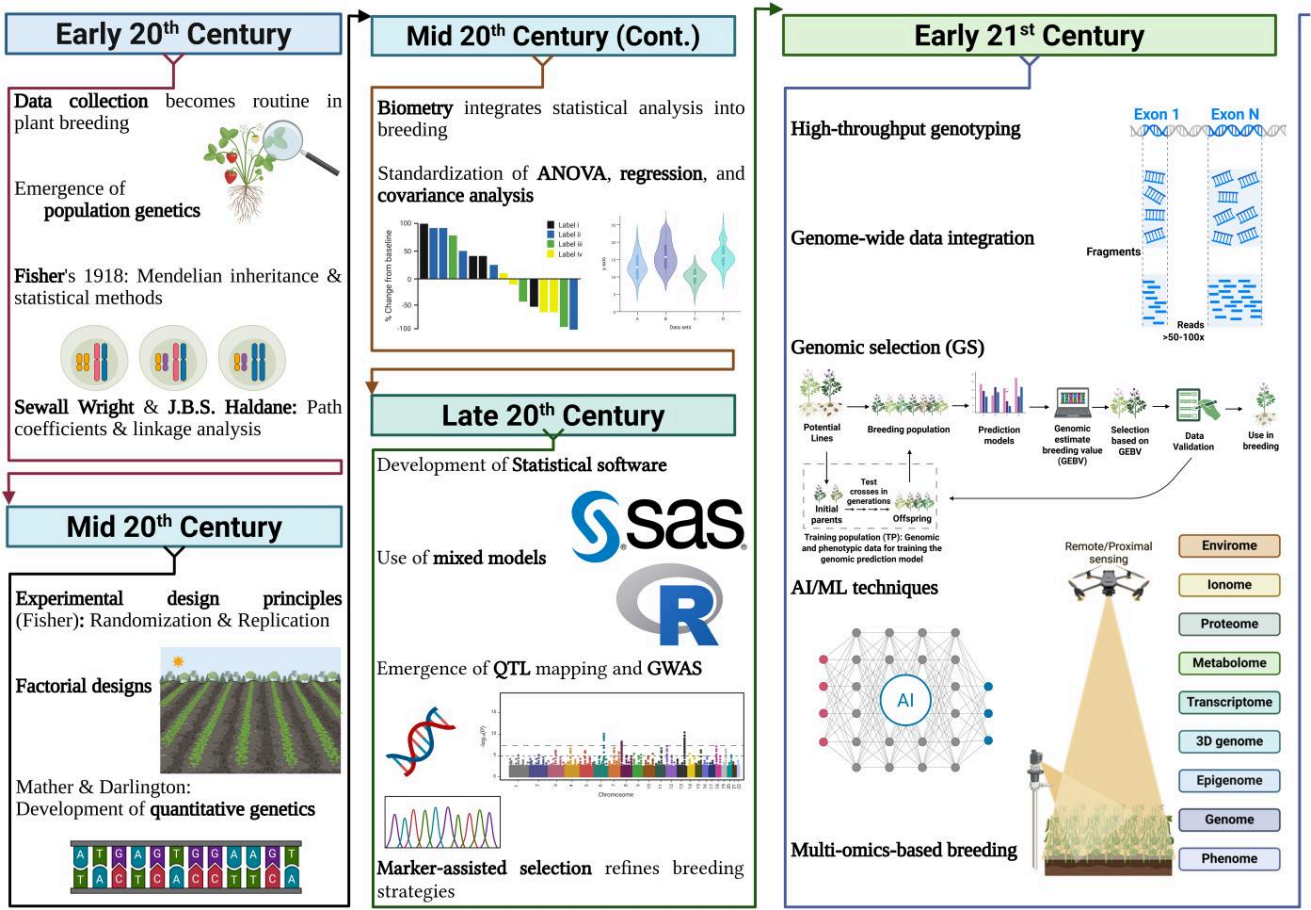
Flowchart of key developments in statistical and computational analyses for plant breeding. QTL, quantitative trait loci; GWAS, genome-wide association study; ANOVA, analysis of variance; AI, artificial intelligence; ML, machine learning. The figure was created using BioRender.com.

Statistics entered the plant-breeding arena when data collection became a routine part of the process, thus representing a paradigm shift, as quantitative methods changed how breeding decisions were made. This integration, which occurred in the early decades of the 20th century, was facilitated by the theoretical framework provided by the early practitioners of population genetics and empirical studies based in agronomy (Cooper et al. 2014). Sir Ronald A. Fisher's 1918 paper on the correlation between relatives under Mendelian inheritance introduced sophisticated statistical techniques to genetics, laying the groundwork for their application in breeding (Reeve and Black 2014). His subsequent development of analysis of variance (ANOVA) and experimental design principles, detailed in “Statistical Methods for Research Workers” (Fisher 1936), provided breeders with robust tools to quantify genetic variance and heritability, key elements of selection strategies (Fig. 2). Alongside Fisher, geneticists such as Sewall Wright and J.B.S. Haldane contributed significantly to the integration of statistics into genetics and subsequently plant breeding (Charlesworth 2017). Wright's path coefficient model, and particularly Haldane's discussions surrounding linkage between genotypes, provided plant breeders with further mathematical frameworks to quantify genetic variance and heritability statistics, which were fundamental to their selection and predictions (Charlesworth 2017).

The integration of statistical genetics took on formal status midway into the 20th century, grounded in the need to better understand quantitative traits controlled by a combination of multiple genes (Elias et al. 2016). Pioneers such as Mather (1943) and Darlington (1958) developed quantitative genetics models that formalized the application of Mendelian genetics within a statistical framework. Using these early models, breeders were able to estimate breeding values and predict outcomes that improved in precision over time. Fisher's innovative experimental design principles (e.g. randomization, control of the experimental environment, and factorial designs) became common practices and allowed for better control over environmental variation in breeding trials (Yates 1964). Factorial designs were a logical implementation of Fisher's experimental design principles which allowed breeders to simultaneously evaluate multiple factors that affected plant traits, thus improving the precision of inferences drawn from experimental data (Elias et al. 2016). Biometrical advancements, including regression and covariance analyses, further refined trait evaluation, allowing breeders to explore genotype–environment interactions critical to successful breeding (Sharma 2006).

The advent of computational tools in the late 20th century amplified these established statistical methods, enabling breeders to tackle larger and more complex datasets (Fig. 2). The introduction of software tools such as SAS (Rodriguez 2011) and R (Ihaka and Gentleman 1996) enhanced the efficiency of multifactorial analyses, building on the foundational work of earlier decades by providing accessible platforms for mixed models, quantitative trait loci (QTL) mapping, and genome-wide association studies (GWAS) (Wang and Xu 2019). These tools supported the analysis of high-throughput genotyping data, integrating phenotypic and genotypic information to refine selection processes (Boer et al. 2007).

With the dawn of the genomics era, plant breeding saw an exponential increase in data availability, requiring even more sophisticated statistical tools (Yoosefzadeh-Najafabadi et al. 2022). High-throughput genotyping allowed for the cost-effective and rapid development of extensive datasets composed of thousands of markers across large populations. This influx of genomic data warranted advanced statistical approaches, such as machine learning (ML) algorithms and Bayesian methods, to extract meaningful relationships between genotypes and phenotypes (Yoosefzadeh-Najafabadi et al. 2022). The integration of high-dimensional statistics became a cornerstone of modern plant breeding through the development of GS, which uses dense marker information to predict the performance of breeding candidates based on statistical models that integrate both phenotypic and genotypic data (Mackay et al. 2019). This approach marked a shift from traditional phenotypic selection to a more precise, data-driven selection strategy, drastically accelerating breeding cycles and improving genetic gains (Mackay et al. 2019).

In the early 21st century, high-throughput phenotyping and multiomics-based breeding programs have revolutionized plant breeding by providing detailed insights and precise strategies. Imaging technologies, such as drones equipped with multispectral cameras, can efficiently evaluate crop health over extensive areas, offering real-time insights into phenotypic variations (Yoosefzadeh-Najafabadi et al. 2023a, 2023b, 2023c). Multiomics approaches combine comprehensive genomics, transcriptomics, proteomics, and metabolomics datasets, enabling more targeted breeding strategies and identifying novel improvement targets with greater precision (Yoosefzadeh-Najafabadi et al. 2023a, 2023b, 2023c). In this domain, advanced statistical methods play a crucial role in harnessing the full potential of high-throughput phenotyping and multiomics data (Fig. 2). ML algorithms, including random forest, support vector machines, and deep learning, are widely employed to analyze large-scale datasets (Yoosefzadeh-Najafabadi et al. 2023a, 2023b, 2023c). These algorithms excel at pattern recognition and predictive modeling, allowing breeders to forecast plant traits such as yield potential from image data collected by drones or multispectral cameras. Moreover, Bayesian statistics help breeders integrate prior knowledge and manage uncertainties in their analyses, providing a robust framework for predicting plant performance and deciphering genetic associations (Yoosefzadeh-Najafabadi et al. 2023a, 2023b, 2023c). This synergy of technology and analytics paves the way for more efficient and resilient crop development.

## The importance of advanced computational methods in plant breeding

The development of advanced computational tools for plant breeding was mainly driven by the increasing complexity and scale of plant breeding programs, in part due to the need to tackle global problems such as food security, the impacts of climate change, and the need to secure biofuels and sustainable agricultural practices. The development of computational tools in the field of plant breeding went through several transformative phases, each characterized by enhancements in data management and analytical capabilities.

With the increasing scope of breeding programs in the 20th century, plant breeders started to explore ways to better managing genetic data. Initially, computational resources were limited to basic statistical analyses carried out on early computers (Afifi and Azen 2014). These early systems were mostly limited to basic statistical analyses, allowing breeders to perform basic calculations for the design of their experiments, analysis of variation in crop trials, and interpretation of Mendelian inheritance across larger data sets. Despite being basic by current standards, these tools established the foundation for integrating more advanced computational technologies.

The sequencing of the *A. thaliana* genome in 2000, along with the genomes of several cereal crops (Fig. 1), paved the way for incorporating genomics in plant breeding. However, the complexity and data volume quickly exceeded the capabilities of manual processing, and breeders needed sophisticated computational-based approaches in order to process and analyze their data (Yoosefzadeh-Najafabadi et al. 2023a, 2023b, 2023c). Bioinformatics emerged to manage sequencing data and provide different methods and algorithms critical for sequence alignment and predicting gene functions. Tools such as BLAST (Camacho et al. 2009, 2023), developed in the 1990s (Lobo 2008), allowed researchers to effectively search large genetic databases for similarities, an important step in identifying genes associated with a trait of interest. The growth of sequence databases, such as GenBank (Benson et al. 2009), enabled comparative genetic analysis across plant species, helping plant breeders better identify markers linked to key traits.

The rise of MAS in plant breeding underscored the need for the rapid processing of genetic markers across diverse lines, making computational efficiency a priority. Breeding programs required systems capable of handling high-throughput genotyping data to identify polymorphic markers associated with phenotypic traits. To support MAS, breeders employed statistical models such as QTL mapping or association mapping (Wang and Xu 2019), which demanded sophisticated computational tools to manage large datasets involved in disentangling the genetics of complex traits. The advent of GS marked a significant advancement in the use of computational tools in plant breeding. GS relies heavily on computational algorithms and ML techniques to process extensive genomic datasets, estimate genetic variances, and predict the breeding values of individual plants (Crossa et al. 2017). The high-dimensional nature of genomic data, along with the need for robust statistical models to handle the inherent noise in biological data, required powerful computing resources and advanced software (Crossa et al. 2017).

While genotyping technology had advanced rapidly, phenotyping remained a bottleneck until recently. High-throughput phenotyping technologies, such as drones with multispectral sensors and ground-based platforms using infrared imagery, generate large volumes of data (Yoosefzadeh-Najafabadi et al. 2023a, 2023b, 2023c). These technologies complement genotyping efforts, but interpreting the resulting high-dimensional datasets requires even more advanced computational tools (Yoosefzadeh-Najafabadi et al. 2023a, 2023b, 2023c). Integrating phenotypic and genotypic data requires robust frameworks for efficient data management and analysis. Consequently, computational advancements have become critical for interpreting images, processing sensor outputs, and synchronizing data from multiple sources, providing actionable insights for plant breeders.

## How does computational biology differ from bioinformatics?

Understanding the differences between computational biology (Box 1) and bioinformatics could help remove some of the confusion surrounding these terms because they are often used interchangeably but are actually distinct in terms of focus, methodologies, and applications. Computational biology merges principles from biology, mathematics, computer science, and physics to model and analyze complex biological systems (Kitano 2002). Computational biology emphasizes computational approaches to solving biological problems by simulating processes, understanding system dynamics, and generating hypotheses for experimental testing. For example, a computational biologist might build a simulation model to study how cells respond to changing environmental conditions or study the dynamics of an ecosystem as it evolves over time. However, this type of research is more hypothesis-driven than the development of algorithms, program coding, or the computational nature of modeling (Kitano 2002; Bartocci and Lió 2016; Chelly Dagdia et al. 2021).

Box 1. What is computational biology? Computational biology refers to the use of computational methods to study biological systems (Kitano 2002). This can include the use of computer models to simulate biological processes and analyze large genomic datasets or the use of different algorithms to identify particular genes of interest (Chelly Dagdia et al. 2021). Computational biology provides the necessary frameworks to study complex biological interactions and predict a system's behavior (Kitano 2002). For instance, in studying metabolic pathways, computational biology allows researchers to explore enzyme interactions and substrate conversions across entire networks rather than focusing on isolated reactions (Nielsen 2017). This systems-level understanding can help identify potential bottlenecks or points of intervention for disease treatment or metabolic engineering in plants. In traditional biology, hypothesis-driven research depends heavily on experimental validation, which can be resource-intensive and time-consuming. However, computational biology can provide a complement to this hypothesis-driven research by allowing researchers to simulate hypothetical scenarios to predict their outcomes prior to experimental execution (Kitano 2002). This capability not only saves time and resources but also enhances experimental design by predicting potential results and refining hypotheses. One example is the use of modeling and simulation to predict how mutations are expected to affect protein structure and function (Stein et al. 2019) to inform research design or predict the pathways leading to disease mechanisms.

By contrast, bioinformatics focuses on the development and use of computational tools to store, analyze, and interpret biological data, particularly the vast amount of data produced by multiomics-based studies (Baxevanis et al. 2020). This approach involves practical data management, including tasks such as sequence alignment, gene finding, and genome assembly. Bioinformatics plays an important role in decoding high-throughput data and can convert complex information to be transformed into meaningful biological knowledge (Thriveni et al. 2024). Modeling problems with biological and genomic data might be a component of bioinformatics but is less of a focus than data analysis and the development of algorithms to process biological data. Specifically, approaches in bioinformatics commonly involve the design of databases, software development for different tasks such as sequence analysis, and statistical modeling to interpret biological findings, with a strong reliance on statistical methods for analyzing and visualizing data (Ma et al. 2020).

Both fields play unique roles in advancing biological sciences. Computational biology often involves dynamic simulations, requiring substantial computational resources to model interactions within cells, organs, or organisms (Kitano 2002; Ijaz et al. 2024). Researchers in this field may employ ML and data mining to validate their simulations against empirical data, focusing on theoretical explorations like predicting the effects of genetic mutations. By contrast, bioinformatics relies on software development and database management (Baxevanis et al. 2020) to facilitate biological discoveries and plays a significant role in omics studies. For example, bioinformatics approaches for genomics studies include assembling genomes, identifying genetic markers, and supporting precision genomics. Both disciplines complement each other, with computational biology offering theoretical insights and bioinformatics providing practical solutions to unravel complex biological questions.

## Leveraging computational biology and bioinformatics in plant breeding

In the area of plant breeding, the convergence of computational biology and bioinformatics has revolutionized the precision with which plant breeders can manipulate and understand the complex biological mechanisms underlying desirable traits. Unfortunately, the distinctions of these fields are often mixed: many plant breeders mistakenly use the terms “computational biology” and “bioinformatics” interchangeably. This conflation can lead to inefficiencies in research and dilute the strategic capability to tackle complex agricultural challenges through targeted breeding programs.

For example, modern plant breeding takes advantage of sequencing technologies such as next-generation sequencing (NGS) and long-read sequencing, which produce a large amount of genomic data (Purugganan and Jackson 2021). Plant genomes are often very large compared with human or mammalian genomes, ranging from hundreds of megabases to tens of gigabases, and they exhibit a diversity of ploidy (e.g. tetraploid durum wheat and hexaploid bread wheat). This complexity, coupled with abundant repetitive sequences and structural variations, poses significant challenges for genome assembly, annotation, and analysis. A single NGS run, for instance, can yield billions of DNA sequencing reads, making manual data processing infeasible. Computational biology utilizes algorithms and software developed for quick genome assembly, homologous sequence recognition, and gene annotation. Tools developed through bioinformatics such as SPAdes (Bankevich et al. 2012) facilitate genome assembly, while BLAST and Clustal Omega (Sievers and Higgins 2014) are important for sequence alignment and homologous sequence identification. Long-read sequencing, now the standard in plant genomics, produces longer DNA reads (up to tens of thousands of base pairs), improving the assembly of these intricate genomes. Current assemblers such as Flye (Kolmogorov et al. 2019) and HiFiASM (Cheng et al. 2021), designed for long-read data from platforms such as PacBio and Oxford Nanopore, have largely replaced older short-read tools, enabling more accurate reconstruction of complex plant genomes.

However, these technologies introduce computational challenges, such as processing high-volume datasets and correcting sequencing errors, especially when working with large-scale plant genomic data. Many of the available bioinformatics tools were created for use with 32-bit systems, generating up to ∼4 billion sequencing reads, making them suitable for small diploid genomes. However, these systems are often insufficient for plant genomes, many of which are beyond this size. This prevents future analyses of structural and functional gene annotation as well as operational traits from the identification of unique genetic markers, and it also prevents breeders from obtaining a comprehensive understanding from large datasets. Therefore, to better analyze such datasets effectively, an efficient workflow is required for cleaning, aligning, and annotating the sequences, which often demand significant computational resources beyond the capabilities of traditional tools. Different approaches are needed to address these challenges, which would make breeding decisions more accurate through bioinformatics.

In terms of computational biology, a profound understanding of GEIs is crucial for developing crops that are well-suited to specific environmental contexts, as these interactions influence plant performance under varying conditions (Yoosefzadeh-Najafabadi and Rajcan 2023). Computational biology provides essential tools to model these interactions accurately, using sophisticated software developed through bioinformatics (Thriveni et al. 2024). Tools such as GEMMA (Zoubarev et al. 2012) and GCTA (Yang et al. 2011) are used to analyze large datasets to make prediction, such as how different wheat genotypes might respond to drought by factoring in environmental variables such as temperature, sunlight, and soil moisture.

From these examples, it is evident that plant breeding is inherently multidisciplinary, demanding diverse expertise. However, many breeders are not fully trained as computational biologists or bioinformaticians, and it is unrealistic for them to fulfill the rigorous criteria of these fields independently within their breeding programs. Instead, breeders utilize these disciplines to accelerate the pace and efficiency of their work. Adopting titles such as “computational biologist” or “bioinformatician” alongside “plant breeder” may not accurately capture their roles, as these terms imply equivalent expertise in each discipline. For plant breeders, the cornerstone of their work is plant breeding, with other techniques serving to expedite this primary objective. Consequently, there is a need for a specific term that accurately reflects the role of plant breeders who integrate computational and bioinformatic techniques without causing confusion with other specializations. This would ensure clarity in defining their unique contribution to the plant breeding landscape.

We can identify different scenarios among plant breeders: (i) those who use results from bioinformatics tools and computational biology within their breeding programs could be termed “modern plant breeders,” as they incorporate new approaches to facilitate their work, often derived from multiomics high-throughput methods, but these breeders do not exclusively work on developing computational and bioinformatic tools; (ii) those who develop packages for data analysis could be called “plant breeders with relevant bioinformatics expertise,” as they can use bioinformatics tools but use the results of computational biology in their breeding programs; (iii) those who integrate various multiomics datasets using different computational biology tools could similarly be termed “plant breeders with computational biology expertise”; (iv) breeders who perform their own coding, analysis, and biological interpretation represent a unique group that might benefit from a new, specific term that accurately describes their comprehensive skill set without misrepresenting their proficiency in either computational biology or bioinformatics.

## The importance of accurate terminology in plant breeding: why it matters

In today's rapidly evolving scientific landscape, where interdisciplinary approaches are paramount to advancement, the precise use of terminology becomes crucial. Misusing titles or merging fields without proper distinction can dilute the expertise and confuse the roles within specialized disciplines. Just as someone using basic mathematical skills in everyday life is not a mathematician, professionals should not adopt titles that misrepresent their engagement with certain fields. This clarity becomes even more pertinent in plant breeding, where the integration of computational biology, bioinformatics, and multiomics demands a nuanced understanding of these distinct areas.

Within plant breeding, the historical association with genetics as “plant breeding and genetic” has broadened to encompass a variety of disciplines. Modern plant breeding goes beyond genetics, drawing on multiomics areas to enhance the breeding process. Despite the significant role of genetics, it is merely one aspect of the multifaceted process involving various disciplines crucial to advancing plant breeding techniques. In this context, the unchecked merging of titles such as “geneticist,” “computational biologist,” or “bioinformatician” with “plant breeder” risks obscuring the specific contributions of each discipline.

A similar caution applies when considering the computational dimension of plant breeding. As the field increasingly relies on computational tools and bioinformatics to manage and analyze large datasets, it is essential that plant breeders focus on what these disciplines can offer without overextending into realms that require distinct expertise. This distinction is critical for guiding future education and training for plant breeders, helping students and professionals appreciate the individual and complementary roles of various fields while maintaining a clear identity as plant breeders.

Therefore, rather than broadly adopting terms from other disciplines, it is more appropriate to refine and create new terms that encapsulate the unique integration of skills and knowledge in plant breeding. This practice not only honors the separate contributions of different fields but also provides clearer communication about the roles and expertise involved in plant breeding. Such precision in language will foster a more accurate representation of multidisciplinary work and aid in the development of future plant breeding experts well-versed in the appropriate uses of computational and biological sciences.

## The role and relevance of computational plant breeding

The term “computational plant breeding” captures the integration of computational methods to enhance the design and optimization of plant breeding programs. Therefore, a computational plant breeder is best understood as a professional who leverages computational biology, data science, bioinformatics, and a wide range of computational tools to expedite and refine the breeding process. This may involve developing software packages to biologically interpret data or integrating AI and other sophisticated algorithms into breeding programs. Nonetheless, while a computational plant breeder utilizes these techniques, they do not necessarily fulfill all the criteria or expertise required in the bioinformatics or computational biology fields. Their primary focus remains on applying such techniques to enhance breeding outcomes.

Importantly, becoming a computational plant breeder does not inherently grant one the title of a bioinformatician or computational biologist unless formal education and training in these areas are pursued. For plant breeders who have undergone dedicated studies in bioinformatics or computational biology, it would be accurate to adopt dual titles like “computational plant breeder and computational biologist” or “computational plant breeder and bioinformatician.” However, when these computational techniques are applied solely within breeding contexts, the title “computational plant breeder” most aptly reflects their role. By recognizing and respecting the boundaries between these disciplines, the term “computational plant breeding” emphasizes the unique contributions and integrations that computational methods bring to plant breeding while also providing educational and career pathways for future breeders.

## Tools for advancing computational plant breeding

Computational plant breeding leverages diverse tools to store, manage, and analyze large datasets and new datasets generated through modern multiomics technologies. These tools include programming languages, data management systems, and analysis platforms available to plant breeders for approaches such as genome assembly, prediction of traits, and multiomics (Table 2). Most programming languages such as R (Ihaka and Gentleman 1996), Python (Van Rossum and Drake 2003), C, C++, and Perl are usually used to handle different analyses such as data preparation, preprocessing, traditional statistical analyses, and ML algorithms. For example, R and Python support statistical analyses and visualization, while C and C++ power high-performance biological software such as HTSlib for handling sequencing data, and Perl remains prevalent for bioinformatics scripting, particularly text processing, data parsing, and automating pipelines. The choice of language depends on the task, ranging from custom algorithm development to leveraging existing tools, rather than inherent superiority, as most modern languages provide robust ML and statistical capabilities.

**Table 2.** Software and tools used for computational plant breeding

Category	Examples	Primary language	Uses for plant breeding	Compatibility with HPC systems
Genome assembly	Flye, HiFiASM, Canu	Python, C	Assemble long-read sequencing data (PacBio, Oxford Nanopore) for complex plant genomes	Yes
Genomic analysis	HTSlib, PLINK, GATK	C, Bash, Java	Sequencing data processing, genotype preprocessing, variant calling for GWAS and selection	Yes
Variant analysis	VCFtools, BEDTools, BCFtools	Bash, C	Manipulate variant call format (VCF) files, analyze genomic features, and integrate datasets	Yes
Statistical modeling	lme4, ASReml-R, BGLR, rrBLUP	R	Mixed-effect models, Bayesian genomic prediction, genomic selection, and breeding value estimation	Partial (ASReml-R, BGLR)
Genomic prediction and GWAS	GAPIT, rMVP, GenABEL, Vcf2gwas	R, Python	Multivariate GWAS, genomic prediction, and genotype–phenotype association analysis	Yes (rMVP, Vcf2gwas)
Machine learning	Scikit-learn, Keras, H_2_O.ai	Python	Predictive modeling, deep learning for phenotype prediction, and trait classification	Yes
Data preprocessing	AllInOne-Preprocessing, Pandas, NumPy	R, Python	Clean and preprocess phenotypic and genomic datasets for analysis	Yes (Pandas, NumPy)
Transcriptomics	DESeq2, edgeR, Bioconductor (RNA-Seq)	R	Differential gene expression analysis, RNA-Seq preprocessing for trait-related gene identification	Partial
Proteomics	MaxQuant, Proteome Discoverer	C++, Various	Protein identification and quantification to study trait manifestation	Yes
Metabolomics	MetaboAnalyst, XCMS	R	Analyze and visualize metabolic profiles to associate metabolites with breeding traits	Partial
Multiomics integration	MOTBX, Omics Fusion	Various	Integrate genomics, transcriptomics, proteomics, and metabolomics for systems biology approaches	Yes
Scripting	BioPerl, Bash	Perl, Bash	Automate bioinformatics pipelines and data manipulation tasks	Yes
Data management/cloud	AWS, Google Cloud, Breedbase	Various	Store, share, and manage multiomics and phenotypic data across collaborative networks	Yes
High-performance computing	SLURM, Torque	Various	Orchestrate parallel processing for genomic predictions, simulations, and large-scale analyses	Yes
Experimental design	Agricolae	R	Design and analyze field trials, perform ANOVA for phenotypic data assessment	No

R packages such as AllInOne-Preprocessing can handle data preprocessing tasks in a user-friendly environment for possible breeding datasets. Agricolae (de Mendiburu and de Mendiburu 2019), lme4 (Bates et al. 2015), ASReml-R (Butler et al. 2017), and GenABEL (Aulchenko et al. 2007) support tasks from experimental design to genetic analysis. Agricolae is tailored for analyzing experimental designs and performing ANOVA, which are essential for assessing the results of field trials (de Mendiburu and de Mendiburu 2019). lme4 and ASReml-R excel in mixed-effect modeling, which is essential for phenotype–genotype dissection in breeding experiments (Bates et al. 2015; Butler et al. 2017). GenABEL and rrBLUP (Endelman 2011) focus on GWAS and genomic prediction, which enhance genetic analyses and selection processes. BGLR (Pérez and de los Campos 2014), rMVP (Yin et al. 2021), and GAPIT (Lipka et al. 2012) further facilitate genomic prediction and multivariate GWAS, supporting breeders in pinpointing genetic markers and predicting breeding values. Python's versatility complements the capabilities of R with libraries such as Scikit-learn (https://scikit-learn.org/stable/about.html#citing-scikit-learn) (Kramer 2016) and Keras (Ketkar 2017) for ML and deep learning, which are essential for phenotype prediction and complex data representation. Pandas (Snider and Swedo 2004) is crucial for data manipulation, while NumPy supports numerical computations vital for handling extensive genomic data (Harris et al. 2020). Vcf2gwas enables GWAS in Python, streamlining the genotype–phenotype association process (Vogt et al. 2022). H_2_O.ai caters to scalable ML tasks, integrating with both R and Python to enhance predictive accuracy (Ajgaonkar 2022). These tools empower breeders to develop advanced models for trait prediction and selection, meeting the demands of modern plant breeding.

In addition to programming languages, Bash scripting and command-line tools like PLINK (Purcell et al. 2007), VCFtools (Danecek et al. 2011), and BEDTools (Quinlan and Hall 2010) are invaluable for automating data management and performing multiomics analyses. PLINK is key for genotype data preprocessing, while VCFtools manages variant data from sequencing projects. BEDTools facilitates genomic feature analysis, integrating genomic and phenotypic data. These tools, coupled with high-performance computing (HPC) systems such as SLURM and Torque, ensure the efficient orchestration of computationally intensive tasks fundamental to breeding programs (Doosthosseini et al. 2024). HPC environments enable the parallel processing of genomic predictions, simulations, and data analyses, supporting the scalability required in large-scale breeding initiatives (Doosthosseini et al. 2024).

For multiomics approaches in plant breeding, there are available packages developed in different languages useful for computational plant breeders. Transcriptomics involves the study of the complete set of RNA transcripts produced by the genome under a specific condition, providing information on gene expression patterns and regulatory mechanisms in the form of differential expression (Brink et al. 2016). Tools such as DESeq2 (Love et al. 2014) and edgeR (Robinson et al. 2010), implemented in R, are popular for differential expression analysis, allowing breeders to pinpoint genes (transcriptomics) relevant to important traits. In Bioconductor, there are also additional tools for preprocessing and normalization of RNA-Seq data, which is important to accurately assess data quality. For proteomics, the software packages MaxQuant (Prianichnikov et al. 2020) and Proteome Discoverer (Orsburn 2021) have been developed to identify and quantify proteins from spectrometric data. This identification process and quantification may ultimately help breeders understand the role of the proteome in the manifestation of traits. For metabolomics, MetaboAnalyst (Xia and Wishart 2016) and XCMS (Domingo-Almenara and Siuzdak 2020) in R provide tools for analyzing and visualizing metabolic data, allowing computational plant breeders to associate specific metabolite profiles with desirable traits. Integrating multiomics datasets offers a holistic view of plant biology, facilitating a systems biology approach to breeding that enhances the precision and efficiency of selection programs. This integration is supported by platforms such as the Multi-Omics toolbox (MOTBX) and Omics Fusion, which combine different omics data to generate comprehensive insights into plant trait development and adaptation strategies (Brink et al. 2016; Alonso-Andrés et al. 2023).

Cloud technology is especially beneficial in global breeding programs, where shared data and insights can significantly expedite the development of improved crop varieties. Cloud-based platforms also provide the computational power necessary to execute complex models and perform data analysis without the burden of maintaining extensive on-premises resources (Bhuiyan et al. 2025). Platforms such as CyVerse (Swetnam et al. 2024) and Google Cloud (Bisong 2019) facilitate breeding programs with the infrastructure to store, manage, and analyze data using dedicated applications optimized for biological research.

## Future perspectives

The field of computational plant breeding is poised to undergo significant progress driven by advancements in technology, data integration, and interdisciplinary collaborations. One key future trend is the increasing use of AI to enhance predictive modeling in plant breeding programs. As these technologies mature, they will provide unprecedented capabilities to analyze complex, multidimensional data from various sources, including genomic, phenotypic, environmental, and management datasets. These advances promise to refine plant selection techniques, optimize breeding strategies, and accelerate the development of crop varieties with desirable traits. Additionally, there is tremendous potential in producing specialized large language models by fine-tuning existing models, such as GPT or BERT, to tailor them specifically for plant breeding programs (Shaikh et al. 2024; Yoosefzadeh-Najafabadi 2025).

Investing in building robust, integrated databases that centralize diverse types of data related to computational plant breeding is highly recommended. Such platforms would not only enhance data accessibility but also improve collaborative efforts across research institutions and geographic locations. Cloud-based infrastructures could facilitate real-time data sharing and collaborative analysis, allowing computational plant breeders to leverage global genetic resources more effectively (Johnraja et al. 2024). These centralized databases should be equipped with advanced analytical tools that can handle the complexity and volume of the data, offering breeders a streamlined way of applying AI algorithms to generate actionable insights.

Furthermore, the next phase in plant breeding will likely involve a deeper integration of multiomics approaches, providing a more comprehensive understanding of plant biology and trait expression. This integrative approach will require the development of novel computational tools and algorithms capable of processing and interpreting complex datasets, which will be vital for identifying causal genes and their interactions (Yoosefzadeh-Najafabadi et al. 2024). Developers of computational plant breeding platforms should prioritize the user-friendly visualization of multiomics data, allowing breeders to discern patterns and relationships that support selection decisions.

The enhancement of phenotyping capabilities through advanced imaging technologies and sensor networks is another future direction for computational plant breeders. High/ultra-throughput phenotyping, using unmanned aerial vehicles, ground-based platforms, and satellite imagery equipped with multispectral and thermal cameras, will provide granular and diverse phenotypic data across large breeding populations (Yoosefzadeh-Najafabadi et al. 2023a, 2023b, 2023c). The integration of these advanced phenotyping methods with genomic data could transform how traits are evaluated and selected, offering insights into genotype-by-environment interactions that were previously challenging to measure (Yoosefzadeh-Najafabadi et al. 2023a, 2023b, 2023c). Therefore, the development of algorithms to process and analyze high-resolution phenotypic data will be crucial for translating these insights into practical breeding outcomes.

The role of precision agriculture in plant breeding is also set to expand, with technology enabling more precise monitoring and management of breeding trials. Implementing precision farming practices will require enhanced computational systems that can manage and analyze data from various field sensors, drones, and satellite imagery (Yoosefzadeh-Najafabadi et al. 2023a, 2023b, 2023c). These tools can monitor environmental conditions and plant responses in real time, providing valuable data that support informed breeding decisions. As these systems evolve, they could facilitate the breeding of crops specifically suited to local environmental conditions and agricultural practices, improving productivity and sustainability.

Looking ahead, it is also imperative to rethink the education and training of future plant breeders. As technology continues to advance, breeding programs will require professionals proficient in both traditional plant breeding techniques and modern data science skills. Curricula should therefore evolve to incorporate training in computational techniques, data management, and advanced statistical methods. Interdisciplinary education that bridges agricultural science, computer science, and bioinformatics will be essential to prepare the next generation of breeders for the challenges and opportunities presented by a data-driven future.

In addition to technical skills, fostering interdisciplinary collaborations will be paramount for tackling the complex, multifaceted challenges facing modern agriculture. Collaborative networks that bring together biologists, computer scientists, agronomists, and environmental scientists can leverage their diverse expertise to innovate and solve problems in plant breeding. Collaborative platforms and initiatives should encourage knowledge exchange and innovation by removing barriers to information sharing and fostering an environment of open science.

Finally, as regulatory and ethical considerations grow in importance, it is essential for the plant breeding community to engage stakeholders in discussions about the societal impacts of using high-tech breeding strategies. Public understanding and acceptance of biotechnology and data-driven breeding approaches will be critical to their successful deployment. Transparent communication, along with regulatory frameworks that ensure biosafety and ethical considerations, can aid in gaining public trust and acceptance.

## Concluding remarks

The integration of computational tools into plant breeding represents a groundbreaking transformation in agricultural science, enabling breeders to navigate and analyze vast, complex datasets effectively. This evolution, which is known as computational plant breeding, capitalizes on advancements in computational biology, bioinformatics, and data science to enhance the precision and efficacy of breeding programs. By adopting a systems-based approach, computational plant breeders can simulate plant growth under diverse conditions, refine trait selection processes, and leverage multiomics data to gain insights into genetic, environmental, and phenotypic interactions. The development and application of this interdisciplinary field involve the strategic convergence of skills from computational biology, bioinformatics, and traditional plant breeding practices. While computational plant breeders employ tools and algorithms from these domains, it is crucial to maintain precise terminology that reflects their unique expertise and contributions, avoiding potential confusion with the distinct fields of computational biology and bioinformatics. Looking forward, the future of plant breeding will likely be characterized by the increased adoption of AI technologies, creating unprecedented opportunities for data integration and analysis (see Outstanding Questions Box). This will require comprehensive educational programs to train the next generation of breeders in both advanced computational techniques and traditional agronomic knowledge. Moreover, fostering interdisciplinary collaborations and engaging with stakeholders will be essential to address societal impacts and promote the ethical deployment of high-tech breeding strategies. As this field evolves, strategic and transparent communication with the public will be crucial to garnering support and trust for the use of these progressive methodologies in plant breeding.

Advances Box

- The introduction of the term “computational plant breeding” offers a precise means to describe the integration of computational biology, bioinformatics, and data science, distinguishing the unique skill set of modern plant breeders.
- Recent advancements in multiomics and high-throughput phenotyping empower breeders to analyze complex genetic, phenotypic, and environmental interactions with unprecedented precision.
- Cutting-edge artificial intelligence approaches are increasingly enhancing predictive modeling, accelerating breeding cycles, and optimizing trait selection in plant breeding programs.
- There is a growing need to update curricula to equip future plant breeders with skills in data analysis and computational tools alongside traditional breeding techniques.

Outstanding Questions Box

- How can AI tools be optimized to boost predictive accuracy in plant breeding?
- What standards can support the integration of multiomics data across breeding programs?
- How can collaborations across biology, computing, and agronomy be strengthened?
- What ethical concerns arise from computational breeding, and how can they be addressed in policy and public outreach?
- How should education evolve to equip future breeders with skills in data science and traditional methods?
- What are the societal impacts of high-tech breeding, and how can public trust be built?

## Data Availability

No data were used for the research described in the article.

## Dive Curated Terms

The following phenotypic, genotypic, and functional terms are of significance to the work described in this paper:

GPT Gramene: AT2G41490
GPT Araport: AT2G41490
